# Decision‐Making Balance in Coproduced Priority Setting for a Parent Carer Empowerment Intervention

**DOI:** 10.1111/hex.70953

**Published:** 2026-10-29

**Authors:** Jim Reeder, Saira Minhas, Erica Breuer, Jane R Smith, Sally Kendall, Christopher Morris

**Affiliations:** ^1^ PenCRU (Peninsula Childhood Disability Research Unit), Health & Community Sciences University of Exeter Medical School UK; ^2^ PenCRU Family Faculty, Health & Community Sciences University of Exeter Medical School UK; ^3^ Department of Medicine and Public Health The University of Newcastle Newcastle New South Wales Australia; ^4^ Exeter Collaboration for Academic Primary Care (APEx) University of Exeter Medical School, University of Exeter UK; ^5^ Centre for Health Services Studies University of Kent UK

## Conflicts of Interest

The authors declare no conflicts of interest.

We thank the letter's authors for their thoughtful comments on our research [1]. It offers a welcome opportunity to reflect more deeply on the complexities of how power is negotiated and exercised in coproduction and, specifically, on the relationship between representation and the processes through which collective research priorities are developed.

Our research sought to develop new ways of working that would support the empowerment of parent carers in healthcare settings, which necessarily involves critically examining existing power dynamics between parent carers and service providers. Importantly, these very power dynamics were also at play in our coproduction workshops, including during the priority‐setting process, and we agree that the decisions made about these processes deserve greater scrutiny.

As we described, our Theory of Change (ToC) working group brought together four parent carers, three allied health professionals (AHPs), one manager and one representative from the commissioning service. The group was assembled using convenience, volunteer and purposive sampling to bring together diverse perspectives from across one service, rather than to achieve numerical parity. The group was co‐facilitated by the lead researcher and a parent carer research partner.

During the prioritisation process, the group spent time in discussion considering which outcomes, groups of outcomes or causal pathways should be prioritised as target areas for change. Each group member then cast two votes, which resulted in the prioritisation of effective two‐way information sharing and trusting relationships between service providers and parent carers. These priorities subsequently informed the intervention's focus on service provider behaviours.

As the letter highlights, the distribution of votes was not numerically equal between parent carers and other group members. Parent carers held 8 of the available 18 votes, while service providers, the manager and commissioner held 10. Votes were cast anonymously, so we did not collect, and cannot now report, the distribution of votes by participant group. We acknowledge that this limits the extent to which readers can scrutinise the prioritisation, and we should have stated in the original article that votes were made anonymously and therefore not analysable by group.

However, this has led us to reflect on whether numerical distribution of votes can provide an adequate account of decision‐making power within a coproduction process. This information would not, by itself, tell us how participant contributions were heard, negotiated, influenced or incorporated into the decisions ultimately made. This is particularly important as the ToC workshops were not designed to collect individual, independent perspectives. The participatory process included structured activities and facilitated discussion between participants with different experiences and forms of knowledge. There were also opportunities for informal interactions, where participants were able to share experiences and perspectives over coffee and/or a meal.

These features are important to our understanding of what the resulting priorities represent. The two outcomes selected cannot necessarily be assumed to represent the views that each participant brought into the room. Rather, they were generated within a particular social and epistemic context, where participants learned about each other's experiences, considered different perspectives and developed shared ideas through both formal and informal interaction and discussion. Perhaps therefore, the question of equity and balance should extend beyond numerical representation to consideration of the epistemic process: the conditions under which people participate, share, question and generate knowledge, and through which interpretations become meaningful and influence what becomes collectively understood and prioritised.

We do not intend this argument to suggest that interaction and collective meaning‐making automatically resolves power differentials. Indeed, the opposite of course may be true. Power can operate through the very processes by which knowledge is shared, and collective priorities are established. Participants may differ in confidence, familiarity with research, professional authority and expectations about whose knowledge is legitimate. Some forms of knowledge may be more readily recognised than others, and institutional or professional authority may continue to shape interactions even when a group is explicitly constituted as an equal partnership [2].

As such, we suggest that rather than solely focusing on numerical balance, researchers should aspire towards creating the conditions where everybody benefits from working together and where relationships are open, honest, trusting and reciprocal [3]. In doing so participants can be supported to collectively contribute to and shape decisions, although it is acknowledged that the extent to which this can be realised is difficult to establish simply by describing the process or its outcomes.

This relates to the letter's suggestion that in a study that seeks to shift a professional‐lay imbalance, it might be helpful to run priority setting in different strands and comparing them before merging. We do appreciate this would help to make any divergence between the groups visible; however, we believe it also represents a different epistemic process than that which we have articulated above. Separate strands would generate parallel positions to be compared, rather than the shared understanding that our workshops sought to build across perspectives. It would also remove the opportunity for participants to learn from one another, which we regarded as central, though we do acknowledge that mixed groups may have exposed parent carers to professional influence and that this is a genuine trade‐off.

We would like to clarify the role of the broader PPIE group mentioned in our limitations. This group comprised five parent carer research partners and were involved across all stages of the study. A parent carer partner co‐facilitated both workshops, and the group contributed to prioritising the intervention focus and shaping its design (Table 2 of our article). We accept that they did not alter the votes cast within the working group, and that our wording could have made their composition clearer, but their involvement did provide an additional opportunity for parent carer perspectives to influence the decisions that followed.

Ultimately, we agree with the letter's authors that in coproduction research, the decision‐making process is itself worthy of examination. We would extend this observation by suggesting that power operates not only through formal decision rules, but through the wider epistemic and relational processes in which decisions are made. The management of power in coproduction is a continuing and, arguably, wicked problem, where any procedural decision has both intended and unintended consequences [4]. There is unlikely to be a single group composition, voting procedure or participatory technique that can guarantee equitable coproduction. Instead, researchers need to remain reflexive about how participation is structured, whose knowledge is being recognised, how relationships shape decision‐making and what consequences follow from collective decisions.

We are grateful to the letter's authors for prompting this reflection. Their comments reinforce for us that, as researchers, we must go beyond asking whether power was equal or unequal in a particular decision and strive to create more equitable epistemic and relational conditions for collective enquiry and decision‐making.

## Author Contributions

**Jim Reeder:** writing – original draft. **Saira Minhas:** writing – review and editing. **Erica Breuer:** writing – review and editing. **Jane R Smith:** writing – review and editing. **Sally Kendall:** writing – review and editing. **Christopher Morris:** writing – review and editing.

## Funding

The authors have nothing to report.

## Data Availability

Data sharing not applicable to this article as no datasets were generated or analyzed during the current study.
